# The promoter of cell growth- and RNA protection-associated SND1 gene is activated by endoplasmic reticulum stress in human hepatoma cells

**DOI:** 10.1186/s12858-014-0025-2

**Published:** 2014-12-11

**Authors:** Sandra Armengol, Enara Arretxe, Leire Enzunza, Sarai Mula, Begoña Ochoa, Yolanda Chico, María José Martínez

**Affiliations:** Department of Physiology, Faculty of Medicine and Dentistry, University of the Basque Country UPV/EHU, Barrio Sarriena s/n, 48940 Leioa, Spain

**Keywords:** SND1 transcriptional activity, Tudor and nuclease domain containing protein 1, Tudor-SN, ER stress response, ATF6

## Abstract

**Background:**

Staphyloccocal nuclease domain-containing protein 1 (SND1) is involved in the regulation of gene expression and RNA protection. While numerous studies have established that SND1 protein expression is modulated by cellular stresses associated with tumor growth, hypoxia, inflammation, heat-shock and oxidative conditions, little is known about the factors responsible for SND1 expression. Here, we have approached this question by analyzing the transcriptional response of human SND1 gene to pharmacological endoplasmic reticulum (ER) stress in liver cancer cells.

**Results:**

We provide first evidence that SND1 promoter activity is increased in human liver cancer cells upon exposure to thapsigargin or tunicamycin or by ectopic expression of ATF6, a crucial transcription factor in the unfolded protein response triggered by ER stress. Deletion analysis of the 5’-flanking region of SND1 promoter identified maximal activation in fragment (-934, +221), which contains most of the predicted ER stress response elements in proximal promoter. Quantitative real-time PCR revealed a near 3 fold increase in SND1 mRNA expression by either of the stress-inducers; whereas SND1 protein was maximally upregulated (3.4-fold) in cells exposed to tunicamycin, a protein glycosylation inhibitor.

**Conclusion:**

Promoter activity of the cell growth- and RNA-protection associated SND1 gene is up-regulated by ER stress in human hepatoma cells.

## Background

The endoplasmic reticulum (ER) mainly functions in proper protein synthesis and maturation, and transport of the correctly folded molecules. When protein folding or export is perturbed and an excess of misfolded client proteins accumulate in the ER lumen (ER stress), eukaryotic cells activate a homeostatic response with a primarily cytoprotective effect, that is collectively termed the unfolded protein response (UPR). The UPR triggers a set of signaling cascades for the control of gene transcription and translation programs, which while emanating from the ER, requires the nucleus and the Golgi apparatus for intracellular signal transduction [1]. In mammals, the principal branches of UPR signaling are mediated by three ER transmembrane stress sensors: PERK (double-stranded RNA-activated protein kinase-like ER kinase), IRE1 (inositol-requiring transmembrane kinase and endonuclease 1) and ATF6 (activating transcription factor 6). Under unstressed condition, these sensors are interacting with the chaperone GRP78 (glucose-regulated protein 78 or BiP) repressing the signaling pathways [2,3]. Upon ER stress, GRP78 is sequestered by unfolded proteins leading to transient and concerted activation of each signaling cascade: PERK-mediated eIF2α phosphorylation, which mediates general translation attenuation during cellular recovery, PERK-derived ATF4/ATF3/CHOP pathway, IRE-1-derived XBP1 mRNA splicing and ATF6 activation (recently reviewed in [4,5]). The three latter pathways induce the expression of distinct but overlapping sets of genes involved in both general and ER-specific proteostasis [3,6-9]. Simultaneously, a subset of cellular mRNAs is translationally silenced by sequestration into discrete cytoplasmic stress granules until stress mitigates or degradation, if ER stress persists [5,10,11].

Staphyloccocal Nuclease Domain containing protein SND1 (also named Tudor-SN, TSN or p100 coactivator) is a multidomain protein that appears to have diverse functions in mammalian cells. Originally described as a transcriptional coactivator essential for normal cell growth [12], SND1 serves multiple functions in biological events spanning from regulation of cell differentiation and proliferation, adipogenesis and biogenesis of lipid droplets to cellular stress responses [13-18]. SND1 has been demonstrated to act as both a nuclease and a ligand. The ample distribution of the protein and its capacity for binding RNA and protein molecules explain the role of SND1 in regulating postranscriptional processes linked to RNA splicing, editing and silencing [18-21]. Accumulating evidence indicates that SND1 plays an important role in RNA protection due to its ability to interact with stress granules protein components and to degrade highly mutated, hyper-edited regions of double-stranded RNAs generated during the cellular stress response [18,22-24]. SND1 also acts as a microRNA (miR) binding protein, having been assigned to bind pre-miR-92a in stress granules and interfere with its maturation under hypoxic conditions [25]. The interaction of SND1 with mRNAs and miRNAs may be of notable relevance in cells undergoing a tumor growth-associated stress because of the potential contribution to angiogenesis regulation. So far, the SND1 overexpression that occurs in multiple types of cancer cells has been interpreted to mean parallel activation of the RNA-induced silencing complex activity and degradation of tumor suppressor mRNAs [26-29].

In the particular case of liver cancer, SND1 and NF-κB intersect at several points. SND1 has been shown to trigger a novel molecular cascade that, mediated by miR-221 and NF-κB activation, leads to induction of angiogenic factors for hepatocellular carcinoma progression [30]. We demonstrated in human hepatoblastoma model HepG2 cells, that the evolutionary conserved SND1 gene promoter is under Sp1 and NF-Y control in basal conditions and under NF-κB functional binding in response to TNFα-mediated inflammatory stress [31]. SND1 mRNA overexpression was also found in TNFα-treated cells [31]. Collectively, these results support the concept that, in liver carcinoma, there is an intertwined relationship between SND1 gene expression and tumour environment inflammation, conditions that are closely linked to ER stress. Here, we seek to extend the previous knowledge of gene transcriptional regulation and have analyzed the transcriptional response of human SND1 gene to pharmacological activation of the UPR in human hepatoma cells. Tunicamycin inhibits protein N-glycosylation [32] and thapsigargin is an inhibitor of the sarco/endoplasmic reticulum Ca^2+^-ATPase pump that disrupts ER calcium homeostasis [33], and both promote accumulation of misfolded or inadequately processed proteins in the ER lumen and cause ER stress. We present first experimental data indicating that promoter activity and expression of human SND1 gene are activated in cells exposed to thapsigargin or tunicamycin and also following ectopic expression of the transcription factor ATF6. Moreover, we have identified the maximal activation in SND/2, a promoter region of 934 nucleotides upstream the transcription start site that contains several putative ER stress response elements (ERSE) for the binding of transcription regulators ATF6, ATF and XBP1.

## Results and discussion

### ER stress activates SND1 gene promoter activity and expression

**Figure 1:**
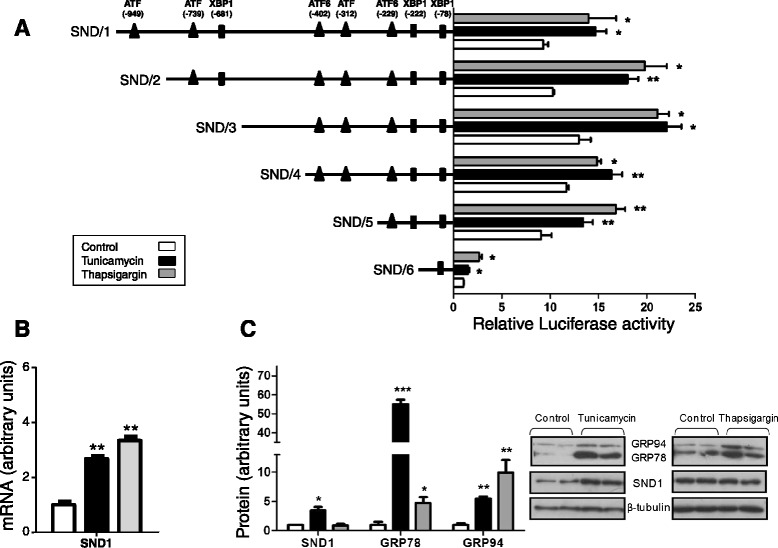
**Activation of human SND1 gene promoter activity and expression by endoplasmic reticulum stress. A)** HepG2 cells were transfected with a luciferase reporter gene driven by six different constructs of the human SND1 gene promoter SND/1-6 and used 24 hours later. Cells were incubated with 5 μg/ml tunicamycin (black) or 1 μM thapsigargin (grey) or the corresponding vehicle (white, control) 2 hours before transfection. Luciferase activity was calculated using a dual luciferase assay and expressed as fold increase relative to the activity of the SND/6 fragment in control cells as described in Methods. **B)** Levels of SND1 mRNA were determined by quantitative real-time PCR in treated and non-treated HepG2 cells and expressed as relative units, setting to 1.0 the value for control cells. **C)** SND1 and chaperones GRP78 and GRP94 protein expression was determined by western blotting using β-tubulin as a loading control and expressed as relative units, setting to 1.0 the value for control cells. Data are presented as mean ± SD from at least three independent experiments. * *P* < 0.05, ** *P* < 0.01, *** *P* < 0.005.

In light of previous reports showing that SND1 participates in a range of stress responses including those associated with cell growth and inflammation, particularly in the liver, and that little is known about the factors controlling SND1 gene expression, we investigated the effect of pharmacologic ER stress on SND1 gene promoter activity in human hepatoblastoma cells. We challenged a human hepatoma cell line, HepG2, with two structurally-unrelated ER stress-inducing drugs, tunicamycin and thapsigargin. Both drugs have proven useful for delineating the molecular grounds of UPR signaling pathways [32-34]. As Fang established [35], we probed 1 μM thapsigargin and 5 μg/ml tunicamycin as the best concentrations for inducing ER stress in HepG2 cells while maintaining cell viability during the 24 h lasting treatment (data not shown). To investigate the response of SND1 promoter activity to these stress inducers, transcriptional activity was measured in HepG2 cells transfected with each of the six 5’ deletion fragments SND/1-SND/6 of the isolated promoter [GenBank: EF690304] that were generated and cloned into the *Firefly luciferase* reporter vector pGL3-Basic. The fragments comprised the promoter regions -1284, +221; -934, +221; -622, +221; -416, +221; -274, +221 and -112, +221. Findings revealed that the activity provided by each luciferase reporter construct increased upon cell exposure to tunicamycin or thapsigargin with rises that oscillated between 50% and 90% depending on the construct (Figure 1A). Maximal activation was detected in fragment SND/2, which covers the promoter region -934 upstream the transcription start site.

To examine whether the response of promoter transcriptional activity to ER stressors was paralleled by increases in the expression of SND1 at the level of mRNA and protein, quantitative real-time PCR and western blot analyses were performed in untreated and ER stress inducers-treated cells. Tunicamycin led to a significant 2.7-fold increase in the SND1 mRNA level (Figure 1B) and 3.4-fold increase in the amount of SND1 protein (Figure 1C). However, whereas there was a 3.3-fold increase in the SND1 mRNA level (Figure 1B) in thapsigargin treated cells, no change in SND1 protein levels were observed (Figure 1C). These findings suggest that each specific form of ER stress promotes differential responses on SND1 translational programs.

The well-established rise in protein levels of the ER marker chaperones GRP78 and GRP94 was used as a surrogate index of the induction of ER stress. We found that the protein levels of chaperone GRP78 in tunicamycin- or thapsigargin-treated cells reached values over 55 and 5 times higher than those measured in control cells (Figure 1C). However, the rise in GRP94 protein was higher in thapsigargin- (10 times) than in tunicamycin- treated cells (5.5 times) (Figure 1C). These findings suggest that the thapsigargin-induced ER calcium loss of homeostasis [33] and the tunicamycin-induced protein N-glycosylation blockage [32] result in activation of the UPR branches of differential intensities, perhaps through accessory interactions, which might condition the selective induction of gene expression and mRNA translation attenuation and stress granules formation [9,36]. Despite this, our findings unambiguously indicate that activation of SND1 gene promoter may be considered one of the processes of cellular adaptability to stressful conditions in HepG2 cells. The finding that SND1 protein is overexpressed in tunicamycin-treated but not in thapsigargin-treated cells suggest that the newly transcribed SND1 mRNA may be fully translated when protein glycosylation is impeded but this protein synthesis is inhibited by thapsigargin-induced ER stress. The latter is not an unexpected finding, in view of that calcium in the ER is required for the initiation of protein synthesis in almost all mammalian cell types, and thapsigargin has been demonstrated to sharply and irreversibly suppress amino acid incorporation within HepG2 [11]. It is conceivable that the primary effect of ER stress is to promote SND1 gen activity and that SND1 protein outcomes depend on the nature, intensity and duration of the stress for fine-tuning the cellular response, with SND1 translation arrest operating when certain ER functional circumstances associated to serious degrees of stress are imposed on the cells.

### Ectopic expression of ATF6 activates SND1 promoter activity

**Figure 2:**
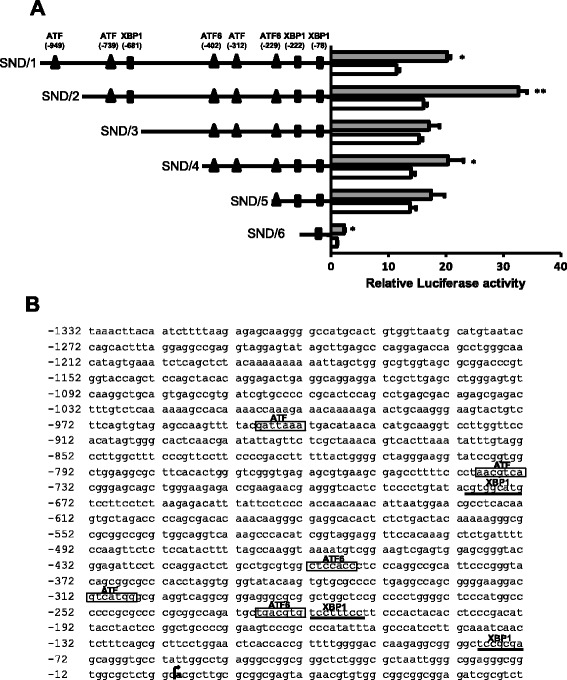
**Ectopic expression of ATF6 transcription factor increases SND1 promoter activity. A)** HepG2 cells were co-transfected with the SND/1-6 constructs of the human SND1 gene promoter and the ATF6 expression vector and used 24 hours later. Luciferase activity was measured in pCGN-ATF6 transfected cells (grey) and mock transfected cells (white, control) and expressed as fold increase respect to the activity of the SND/6 fragment in control cells. Data are presented as mean ± SD from at least three independent experiments. * *P* < 0.05, ** *P* < 0.01. **B)** Nucleotide sequence of human SND1 gene proximal promoter [GenBank: EF690304]. The transcription start site (+1) is shown in bold and marked by an arrow. Boxes indicate putative binding motives for ATF6 or transcription factors from CREB/ATF or EBOX/ATF subfamilies, and XBP1 binding sites are underlined.

To further study the stress-induced activation of the SND1 transcriptional activity, we evaluated the potential role of the transcription factor ATF6 on SND1 promoter activity. ATF6 is initially synthesized and retained by GRP78 in the ER. Upon ER stress, ATF6 is released from GRP78 and deliver to the Golgi apparatus where it undergoes proteolytic processing, and the liberated N-terminal cytosolic fragment ATF6(N) moves then into the nucleus to activate target genes [5]. For that, plasmid-mediated ectopic expression of full-length ATF6 was induced in HepG2 cells transfected with each of the six 5’ deletion fragments of the SND1 promoter and luciferase activity was measured and compared with that shown by mock transfected cells expressing basal levels of ATF6. We observed that luciferase activity of the SND/3, SND/4, SND/5 and SND/6 promoter fragments was either unaffected or minimally activated by ATF6 expression (Figure 2A). However, transcriptional activity of the SND/2 fragment increased by 100% in pCGN-ATF6 transfected HepG2 cells as compared with that measured in mock transfected cells (Figure 2A). Notably, not only the percentage of increase was similar to that caused in the promoter fragments activity by cell exposure to thapsigargin or tunicamycin, but also maximal activation was consistently detected in the SND/2 construct covering the promoter region (-934, +221) (Figure 2A). Bioinformatic analysis revealed that region SND/2 contains all the potential motives for ATF6 and XBP1 binding identified by TESS [37], Jaspar [38] and MatInspector [39] tools along the proximal promoter sequence of SND1 gene (GenBank: EF690304), with the exception of ATF at position -949 (Figure 2B). ATF6 can bind to several *cis*-acting response elements, namely, ERSE (CCAAT-N9-CCACG), ERSE-II (ATTGG-N1-CCACG), and UPR element (TGACGTGG/A) [8]. ATF6 has been identified as the CCACG-binding protein while the general transcription factor NF-Y constitutively occupies the CCAAT/ATTGG part of ERSE and ERSE-II [8]. In previous studies, we demonstrated that the human SND1 promoter lacks TATA box and contains GC boxes and two inverted CCAAT boxes for the functional binding of Sp1 and NF-Y, respectively [31]. We also demonstrated that NF-Y binding at positions -28 and -61 within the proximal promoter is crucial for the basal transcription of SND1 gene [31]. These NF-Y binding sites are located quite far from the predicted stress response elements to be considered as a part of them according to *in silico* analysis. However, it has to be considered that the ERSE motives can be recognized by XBP1 and other transcription factors members of the CREB/ATF and EBOX/ATF subfamilies and the basic-region leucine zipper family [8]. Therefore, more work will be required to identify which of the transcription factors, ATF6 or XBP1 or others, and how they act to regulate the activation of SND1 promoter, and to better characterize the branch of the UPR governing the expression of SND1 gene.

The impact of each UPR branch on liver function in pathologic states has been discussed by Malhi and colleagues, who described the existence of a variety of crosstalks between ER stress, inflammatory response and activation of NF-κB in which ATF6 and XBP1 signaling are involved [40]. We have recently demonstrated that SND1 promoter transcriptional activity increases in response to TNFα-induced inflammatory stress via binding of NF-κB transcription factor at positions -174 and -116 [31]. Upon such inflammatory stress, a 14-fold induction in the ER chaperone GRP78 has been measured in TNFα-treated cells as compared with a negative control (data not shown) supporting the occurrence of perturbations in the ER functional status that could be modulating SND1 gene transcription. Nevertheless, since ATF6 does not substantially affect activity in SND/3-6 and NF-κB mediates its action through -174 and -116 binding sites, SND1 gene expression activation by these agents might be well orthogonal.

## Conclusions

**Figure 3:**
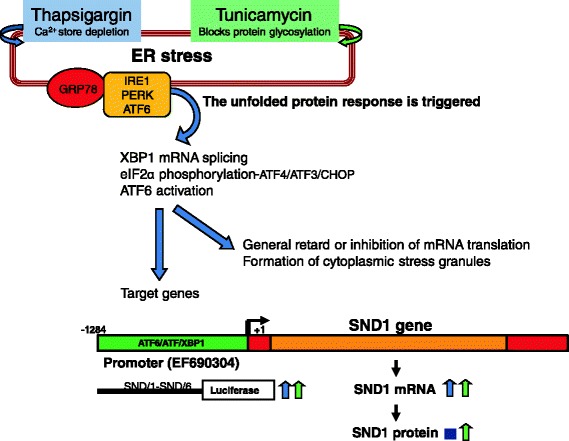
**Scheme illustrating endoplasmic reticulum (ER) stress-mediated activation of SND1 promoter activity.** SND1 is an ER stress target gene. The three families of signal transducers IRE1, PERK and ATF6 sense the protein folding conditions of the ER and transmit that information, resulting in production of transcription regulators that enter the nucleus to drive transcription of UPR target genes. The ER chaperone GRP78 is normally bound to these ER stress sensors and keeps them inactive. By blocking protein N-glycosylation and disturbing Ca^2+^ homeostasis in the ER, tunicamycin and thapsigargin promote accumulation of misfolded proteins that sequester GRP78, leading to the concerted activation of the three stress sensors, which work alone or together to activate UPR target genes. Simultaneously, mRNA translation is globally inhibited and a subset of cellular mRNAs is translationally silenced by sequestration into stress granules. SND1 promoter activity is enhanced by thapsigargin, tunicamycin or ectopic expression of ATF6.

In conclusion, though the exact function of SND1 in mammals is yet to be revealed, our findings identify ER stress as a previously unappreciated up-regulating factor for SND1 gene expression (see Figure 3 for a graphical representation of major findings). We encounter for the first time a direct response of SND1 gene promoter activity to pharmacological ER stress and the potential participation of ATF6 in the ER stress-associated transcriptional activation of the human SND1 gene.

## Methods

### Cells culture and treatment

The HepG2 human hepatocellular carcinoma cells (ATCC, Manassas, VA, USA) were maintained under an atmosphere of 5% CO_2_ at 37°C in EMEM media (ATCC) supplemented with 10% (v/v) foetal bovine serum (ATCC), 2 mM L-glutamine, 100 U/ml penicillin and 100 μg/ml streptomycin (Sigma–Aldrich, St. Louis, MO, USA). Cells were seeded in 96-well plates (12 × 10^3^ cells per well) for the reporter assays and in 6-well plates (7 × 10^5^ cells per well) for mRNA and western blot analysis and were grown to 60-80% confluence. Cells were incubated for 24-26 h with 5 μg/ml tunicamycin or 1 μM thapsigargin (Sigma) and the same volume of the corresponding solvent, methanol or ethanol, was added to the control cells.

### Transfections and luciferase reporter assay

Two hours before transfection, cells received tunicamycin, thapsigargin or the same volume of the corresponding vehicle. Then, cells were transfected using X-tremeGENE 9 transfection reagent (Roche Applied Science, Mannheim, Germany) and received 0.1 μg of the appropriate SND1 promoter reporter vector and 0.1 μg of *Renilla luciferase* pRL-TK (Promega, Madison, WI, USA) as internal control for transfection efficiency. We used six 5’ deletion fragments SND/1-SND/6 comprising the SND1 promoter regions -1284,+221; -934,+221; -622,+221; -416,+221; -274,+221 and -112,+221 cloned into the *Firefly luciferase* reporter vector pGL3-Basic (Promega) as described previously [31]. When indicated, cells were additionally cotransfected with 0.1 μg of the expression plasmid pCGN-ATF6 (Addgene, Cambridge, MA, USA). Mock transfections with the corresponding empty vector were carried out in all cases. After 24 h, cells were lysated and luciferase activity measured using the Dual-Luciferase Reporter Assay System (Promega) in a Synergy™ HT Multi- Detection Microplate Reader (BioTek Instruments Inc, Winooski, VT, USA). *Firefly luciferase* activity from promoter constructs was normalized to *Renilla luciferase* activity. Assays were performed in triplicate and luciferase values were expressed as relative luminescence units, setting to 1.0 the value for SND/6 luciferase activity.

### RNA extraction and quantitative real-time PCR analysis

Total RNA was extracted from HepG2 cells twenty four hours after tunicamycin or thapsigargin treatment using TRIzol reagent (Invitrogene Life Technologies, Barcelona, Spain) according to the manufacturer’s instructions. First strand cDNA was synthesized from 1 μg RNA (NanoDrop ND-1000 spectrophotometer, NanoDrop Technologies, Wilmington, DE) using the SuperScript III system (Invitrogen) and PCR analysis was conducted by the SYBR Green (Applied Biosystem, Foster City, CA, USA) method. Data are expressed as relative expression level and are calculated from the Ct values applying calibration curves and normalized with α-actin, glyceraldehyde 3-phosphate dehydrogenase and TATA box binding protein by using GeNorm 3.5 software [41], as described earlier [42]. The GeneBank accession numbers and primers sequences are: SND1, NM_014390, forward: GTGATCAGATACCGGCAGGATG, reverse: TCTTAATAGCTCTGGCCTCTGCAG; α-actin, NM_001101.3, forward: GAGCACAGAGCCTCGCCTTTGCC, reverse: CGAGCGCGGCGATATCATCATCC; glyceraldehyde 3-phosphate dehydrogenase, NM_002046.3, forward: GGTGAAGCAGGCGTCGGAGG, reverse: GAGGGCAATGCCAGCCCCAG; and TATA box binding protein, NM_003194.3, forward: TTGCAGTGACCCAGCAGCATCAC, reverse: AACCCTTGCGCTGGAACTCGTC.

### Immunoblotting

After 24 h of tunicamycin or thapsigargin treatment, HepG2 cells were washed and lysated. Briefly, proteins (10 μg) from HepG2 cell lysates were resolved by 9% SDS-PAGE at 170 V for 1 h and transferred to polyvinylidene difluoride membranes by semidry transference (1 hour at 20 V). SND1 was detected by immunoblot analysis using antibodies anti-rat SNDp102 that recognize human SND1 [17] and a peroxidase-coupled goat anti-rabbit IgG (Sigma). The level of GRP78 and GRP94 was determined as a marker of ER stress using mouse anti-KDEL IgG (Merck Millipore, Germany) that recognizes the peptide sequence SEKDEL of GRP78 and GRP94. For normalization, β-tubulin was detected by using mouse anti β-tubulin antibody (Santa Cruz Biotechnology Inc., Dallas, TX, USA) and secondary horse anti-mouse IgG. Protein bands were detected using ECL (GE Healthcare Life Sciences, UK) and quantified by optical densitometry using QuantityOne software (Bio-Rad Laboratories, Hercules, CA, USA), as described previously [43]. After normalization, results were expressed as relative units, setting to 1.0 the value for control cells.

### Statistical analysis

The results are shown as the mean ± SD of *n* independent experiments. Statistical significance was assessed using the 2-tailed unpaired Students’ *t*-test, and is denoted * *P* < 0.05, ** *P* < 0.01, and *** *P* < 0.005.

## Abbreviations

ATF6: Activating transcription factor 6
CHOP: C/EBP homologous protein
eIF2α: Eukaryotic initiation factor 2 alpha
ER: Endoplasmic reticulum
ERSE: ER stress response element
GRP78: Glucose-regulated protein 78
GRP94: Glucose-regulated protein 94
IRE1: Inositol-requiring transmembrane kinase/endonuclease protein-1
miR: microRNA
PERK: Protein kinase RNA-like ER kinase
SND1: Staphylococcal nuclease and Tudor domain-containing protein 1
UPR: Unfolded protein response
XBP1: X-box binding protein 1
